# The routine use of skin traction in patients with femoral neck fractures awaiting arthroplasty: a narrative review

**DOI:** 10.1530/EOR-2024-0149

**Published:** 2026-03-02

**Authors:** Ismail Ravat, Allan Roy Sekeitto, Laine Alexander, Nkhodiseni Sikhauli, Lipalo Mokete, Jurek R T Pietrzak

**Affiliations:** Arthroplasty Unit, Charlotte Maxeke Johannesburg Academic Hospital (CMJAH), University of the Witwatersrand, Johannesburg, South Africa

**Keywords:** skin traction, femoral neck fractures, neck of femur fractures, skin wounds, nursing care, arthroplasty

## Abstract

- Femoral neck fractures remain a significant challenge in orthopaedic surgery, particularly among elderly patients.
- This review synthesizes the current peer-reviewed literature on initial management strategies, with a particular emphasis on the use of skin traction.
- Skin traction, which involves the application of adhesive tape, a crepe bandage, and a calibrated pulley system with precise weights, is analysed in terms of its efficacy in clinical practice.
- The review discusses the benefits and drawbacks of skin traction, drawing on recent studies to assess its role in fracture management.
- The goal is to provide nuanced scientific insights into the ongoing discourse surrounding the management of femoral neck fractures.

## Introduction

Femoral neck fractures (FNFs) remain a significant clinical problem to the orthopaedic surgeon, with more than 250,000 cases (1) reported in the United States of America (USA) every year with the number projected to double by the year 2050 (2). Most of these fractures occur in the elderly, with an average age of 72 years and with 75% occurring in women (2). The reported lifetime risk of sustaining a hip fracture remains high with a range of 40–50% in females, compared to 13–22% in males (3). Due to the growing geriatric population driven by increased global life expectancy (4), and fuelled further by increased demands expected on the elderly (5), the incidence of FNFs is antipcipatted to rise. FNFs have been declared as the ‘unsolved fracture’ owing to the high rate of complications and morbidity risk resulting in a significant socio-economic burden (6).

Taking into consideration factors such as ageing global population (4), increased demands on the elderly, and population growth, the incidence of FNFs is projected to increase from 1.6 million people per year in 1990, to around 2.6 million people in 2025, and to 6.3–6.5 million by the year 2050 (7). Globally, it is estimated that 1.3 million people sustain FNFs (8). With reported numbers of annual hip fractures of 100,000 in the UK, 135,000 in Germany, and 18,500 in the Netherlands (4), this poses a serious socio-economic impact and significantly challenges orthopaedic surgeons. Mortality rates associated with FNFs vary from 10% at 30 days to approximately 30% at 1 year post-operatively (6).

FNFs are associated with a significant mortality rate, averaging 10% within the first 30 days post-injury and ranging from 14 to 36% within the first year (4). In a Sub-Saharan African academic institution, the overall 30-day mortality rate for displaced intra-capsular FNFs was 8.01% (8). Although this rate is notably lower when compared to the 10% reported in developed countries (4), it should be noted that in the Sub-Saharan setting, the average waiting-time from admission to surgical intervention, in the form of either hemiarthroplasty or total hip arthroplasty, was 7.9 or 7.61 days, respectively (8), where most guidelines’ gold standard recommendation is early surgical intervention preferentially within 48 h (6). This delay in surgical intervention may adversely impact the outcome of FNFs.

The initial management of FNFs continues to be controversial and evokes intense debate (3, 9, 10). Following fracture, high-grade local groin pain associated with external rotation and shortening of the affected limb is observed, with pain management regarded as important but challenging during this period. Skin traction, utilizing an adhesive tape with crepe bandage placed on either side of the affected limb through a pulley system with appropriate weights, has been regarded as an effective means to relieve pain perioperatively (11). Although nursing care plays an immense role in enhancing patient comfort, alleviating pain, and enhancing general well-being and hygiene (12), prolonged hospital stay and immobilization negatively affect the comfort and well-being of these patients (2). In addition, being confined to bed for prolonged time may lead to other complications, such as pressure sores, infection, thromboembolic pulmonary complications, and delirium (5).

Given the controversy surrounding the initial use of skin traction in managing FNFs, we conducted a comprehensive evaluation of its efficacy by systematically reviewing the literature across databases to determine its usefulness.

## Materials and methods

The use of skin traction in the preoperative or emergency management of FNFs remains a topic of debate. We sought to evaluate this by conducting a narrative review using the following keywords, and Medical Subject Headings (MeSH) terms were used in various combinations to retrieve relevant literature: skin traction, FNFs, preoperative management, emergency orthopaedic care, hip fracture stabilization, skin complications, leg wounds, leg ulcers, and nursing management. Boolean operators (AND and OR) were used to refine the search, for example (“skin traction” AND “hip fracture in elderly”) OR (“intracapsular FNFs” AND “skin complications”).

To identify relevant studies for this review, a comprehensive literature search was conducted across multiple medical and orthopaedic databases, including PubMed, Google Scholar, Scopus, Embase, and Cochrane Library. The search aimed to include studies published within the last 30 years (1990–2024) to ensure the inclusion of both historical and recent developments in the field. The initial search yielded 321 articles. Three independent authors systematically screened the articles, first by reviewing titles and abstracts for relevance, followed by full-text assessments to ensure alignment with our inclusion criteria. Through a process of elimination based on study design, clinical relevance, and data quality, 84 articles were selected for inclusion in this narrative review.

The reasons for exclusion were as follows:

- Duplicates (*n* = 45) – articles that were indexed multiple times across different databases.
- Irrelevant articles based on title/abstract (*n* = 102) – studies that did not focus on skin traction in FNFs or were unrelated to preoperative management.
- Studies having methodological issues (*n* = 55) – studies with small sample sizes, lack of control groups, or unclear methodologies.
- Articles that do not have full text available (*n* = 20) – articles where only abstracts were accessible, preventing full assessment.

Inclusion criteria for the final 84 studies were as follows:

- Studies specifically investigating skin traction in preoperative or emergency management of FNFs.
- Studies that included clinical data, systematic reviews, or comparative studies evaluating the effectiveness of skin traction.
- Research that provided measurable outcomes relevant to surgical preparation, pain control, or fracture stabilization.

**Figure 1:**
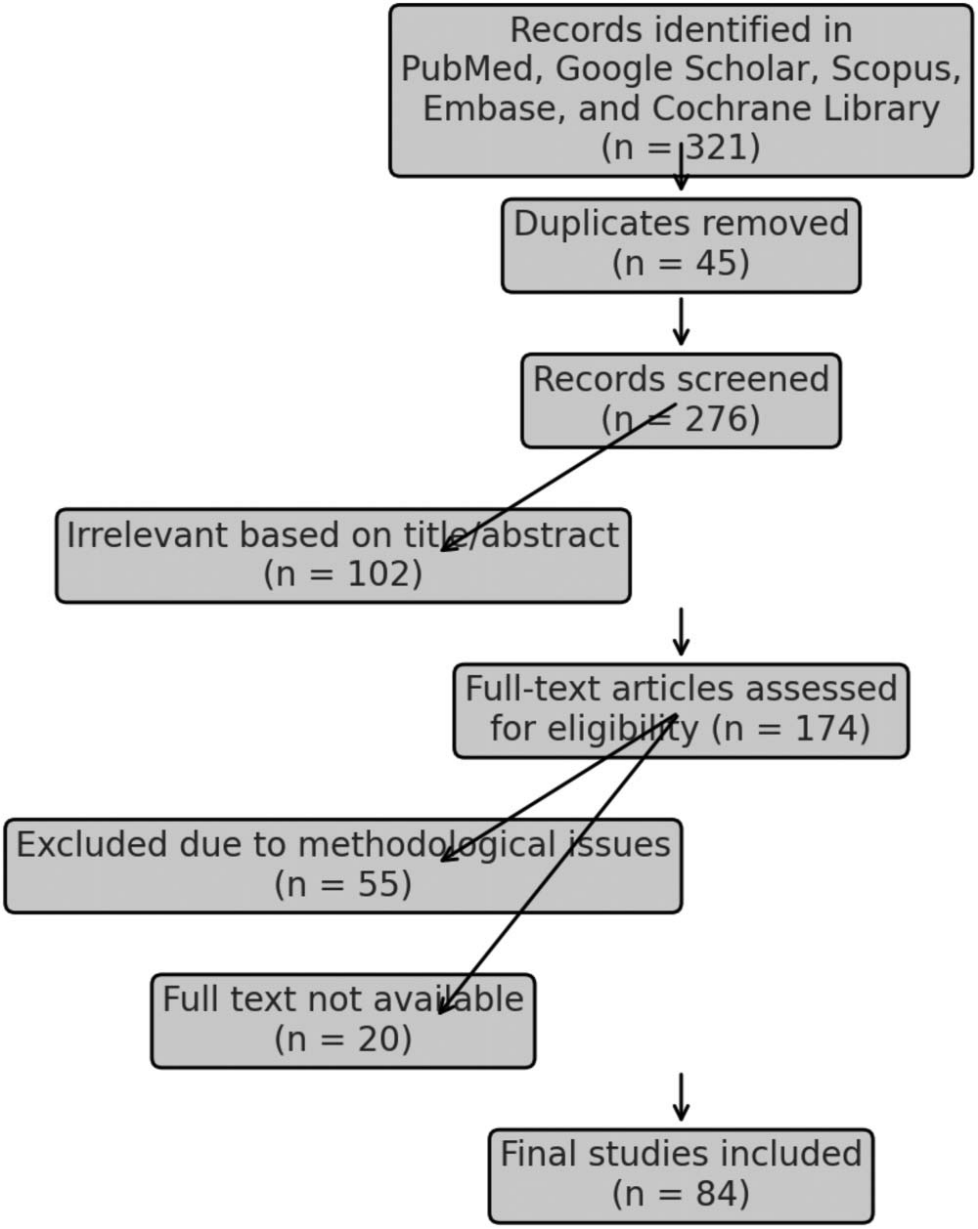
PRISMA flowchart of study selection process.

This structured selection process ensured that only the most relevant and methodologically sound articles were included in the final review (Fig. 1).

As this is a narrative view, we did not apply formal grading systems, such as GRADE (Grading of Recommendations Assessment, Development, and Evaluation) or the Cochrane risk-of-bias tool. Nonetheless, methodological quality was considered throughout study selection and interpretation. Of the 321 articles initially identified, studies were excluded if they demonstrated major methodological weaknesses (e.g. inadequate control groups, very small sample sizes, or incomplete data reporting). This left 84 studies for final inclusion, comprising randomized controlled trials, prospective and retrospective observational studies, and narrative reviews, with sample sizes ranging from small series (<30 patients) to larger controlled cohorts (>200 patients). Among the included literature, common limitations such as heterogeneity in fracture subtypes and inconsistent outcome reporting were noted, and findings from such studies were interpreted with appropriate caution.

By adopting this approach, we aimed to synthesize the most reliable and clinically meaningful evidence available, while acknowledging the inherent variability and risk of bias present within the literature on skin traction for FNFs.

To aid clarity, a summary table has been included outlining the key studies, their design, primary outcomes, and main conclusions regarding the role of skin traction in FNFs (Table 1).

**Table 1:** Summary of key studies evaluating skin traction in FNFs.

Study	Study design	Sample size	Intervention	Outcomes measured	Main findings/conclusions
Anderson *et al.* (28)	RCT	120 patients with proximal femur fractures	Skin traction vs no traction	Pain scores, ease of nursing care, complications	No significant difference in analgesic use or pain scores; skin traction not beneficial
Jerre *et al.* (29)	RCT	100 patients with hip fractures	Preoperative skin traction vs bed rest	Analgesic requirement, complications	No difference in pain relief; traction not recommended routinely
Yip *et al.* (10)	PCS	90 patients	Skin traction vs bed rest	Pain, ease of surgery, complications	No significant differences; recommended discontinuation of routine traction
Endo *et al.* (9)	RCT*	81 patients	Skin traction vs no traction	VAS pain, fracture reduction, operative ease	No effect on pain relief or fracture reduction regardless of waiting time
Munireddy *et al.* (3)	PRS	100 patients >60 years	Skin traction vs pillow elevation	VAS pain	Pillow elevation provided better pain relief; no benefit of skin traction
Hussain *et al.* (30)	RCT	100 patients (hip fractures)	Skin traction vs no traction	Pre-op VAS pain	No significant difference in pain scores; traction not recommended
Shaikh *et al.* (11)	PCS	60 patients with hip fractures	Skin traction vs no traction	VAS pain at 24 h and pre-op	Initial pain reduction observed at 24 h, but no sustained effect; limited benefit
Biz *et al.* (12)	CRS	3 institutions, elderly patients	Skin vs skeletal traction	Nursing care, skin complications, patient comfort	Highlighted challenges with nursing care and increased risk of skin damage with traction
Shabat *et al.* (25)	CS	10 patients with hip fractures	Skin traction	Severe skin sloughing	Reported serious skin complications; recommended caution in frail or vascular-compromised patients

* Single institution.

PCS, prospective comparative study; PRS, prospective randomized study; CRS, cross-sectional study; and CS, case series.

### Mechanism of injury

FNFs account for approximately 50% of fractures around the hip, with around 3–10% affecting individuals younger than 64 years of age (7). In the elderly, low-energy trauma is the most common mechanism of injury and can involve either direct or indirect causes (13). Direct causes involve a fall onto the greater trochanter or forced external rotation of the lower extremity (2, 13). This results in the impingement of the osteoporotic neck onto the posterior lip of the acetabulum. Indirect causes result when muscle forces overwhelm the strength of the femoral neck, resulting in fracture (13). High-energy mechanisms account for FNFs in both the elderly and younger individuals and occur following motor vehicle accidents or following a fall from a significant height (13). Fractures may also result following cyclical loading stresses as seen in athletes, military recruits, and ballet dancers (2). Patients with osteoporosis and osteopenia are at a high risk of insufficiency fractures (13).

In addition to the fragile nature of bone in the elderly, and their subsequent risk of pathological fractures after fall and higher mortality rate (6), advancing age causes intrinsic changes in the skin, leading to thinning, loss of dermal appendages, and increased fragility, which heighten the risk of injury to the skin and hinder wound healing (14).

### Clinical evaluation and initial management of FNFs

Regardless of the mechanism of injury, FNFs constitute an orthopaedic emergency (4) and these patients are assessed and subjected to standard advanced trauma life support (ATLS) principles, followed by a thorough secondary survey (15). Patients with displaced FNFs are typically non-ambulatory with shortening and external rotation of the lower extremity and complain of groin and thigh pain and inability to bear weight on the affected limb. Radiological diagnosis with an AP X-ray of the hip is sufficient to diagnose FNFs. If an X-ray cannot be performed in order to confirm diagnosis, a computed tomography scan is recommended (16). Once diagnosis is confirmed, perioperative management including adequate pain management is mandatory. The National Institute for Health and Care Excellence (NICE) on hip fracture management updated its clinical guidelines and quality standard in 2017 (17, 18). NICE recommendations are based on systemic reviews of the best available evidence and recommend that analgesia be administered immediately on presentation to the hospital, and continued hourly until pain is settled in the ward (19). Not only is sufficient administration of analgesics humane, but it is an essential component in the prevention of delirium in the elderly (16). It is advised to offer non-NSAID analgesia, such as paracetamol every 6 h, unless contraindicated, and if paracetamol alone is not adequate, then oral or intravenous opioids may be titrated according to the patient’s response to pain (19). If there is insufficient response to paracetamol and opioids, then femoral nerve blocks may be considered (16).

A multidisciplinary team approach, combined with a comprehensive geriatric assessment (17), should be implemented to ensure medical stability, optimize surgical fitness, and identify treatable geriatric conditions in elderly patients (16). It is advised that an orthogeriatric unit assess the patient prior to surgery, as these patients often have multiple comorbidities (4).

A urine catheter is inserted, and since FNF patients often experience dehydration and blood loss of up to 500 mL (4), intravenous hydration may be required, with the quantity determined by clinical judgement and careful volume status monitoring (16). A flow rate of up to 100–200 mL/h for isotonic crystalloids is considered safe (19) in patients post-injury and in those with a sustained FNF.

Routine laboratory tests should be conducted for all patients. These tests should include a complete blood count, inflammation markers, INR, partial thromboplastin time, and a basic metabolic profile (16).

It is recommended to perform surgery on the day of admission or the day after admission (17, 19); however, time to surgery varied between 8 and 19 days for hemiarthroplasty and 7–61 days for total hip arthroplasty in a Sub-Saharan Academic Hospital (8). Delay in surgery beyond 24 h is associated with complications such as pulmonary embolism, pneumonia, deep vein thrombosis, urinary tract infections, pressure sores, and dementia (16), hence predisposing to additional morbidity. In addition, the elderly are subject to stress-related gastric mucosal damage and upper gastrointestinal bleeding (20). Chuene *et al.*, therefore, recommended routine perioperative prescription of proton pump inhibitors in elderly patients that sustain FNFs (20).

The goals of preoperative management of patients with FNFs are to limit pain and minimize bleeding in the form of a traction splint application notably skin traction (15), regardless of whether the fracture is open or closed. The femur is associated with extensive muscle damage due to spasm and overriding of fractured bones, with the potential of causing more soft-tissue injury, haemorrhage, neurovascular compromise, and pain (15). The use of skin traction splints has been historically employed in order to help mitigate these factors, as most surgeons believe that the main theoretical advantage of skin traction is to limit pain at the fracture site, by relieving muscle spasm while the patient awaits surgery (10).

**Figure 2:**
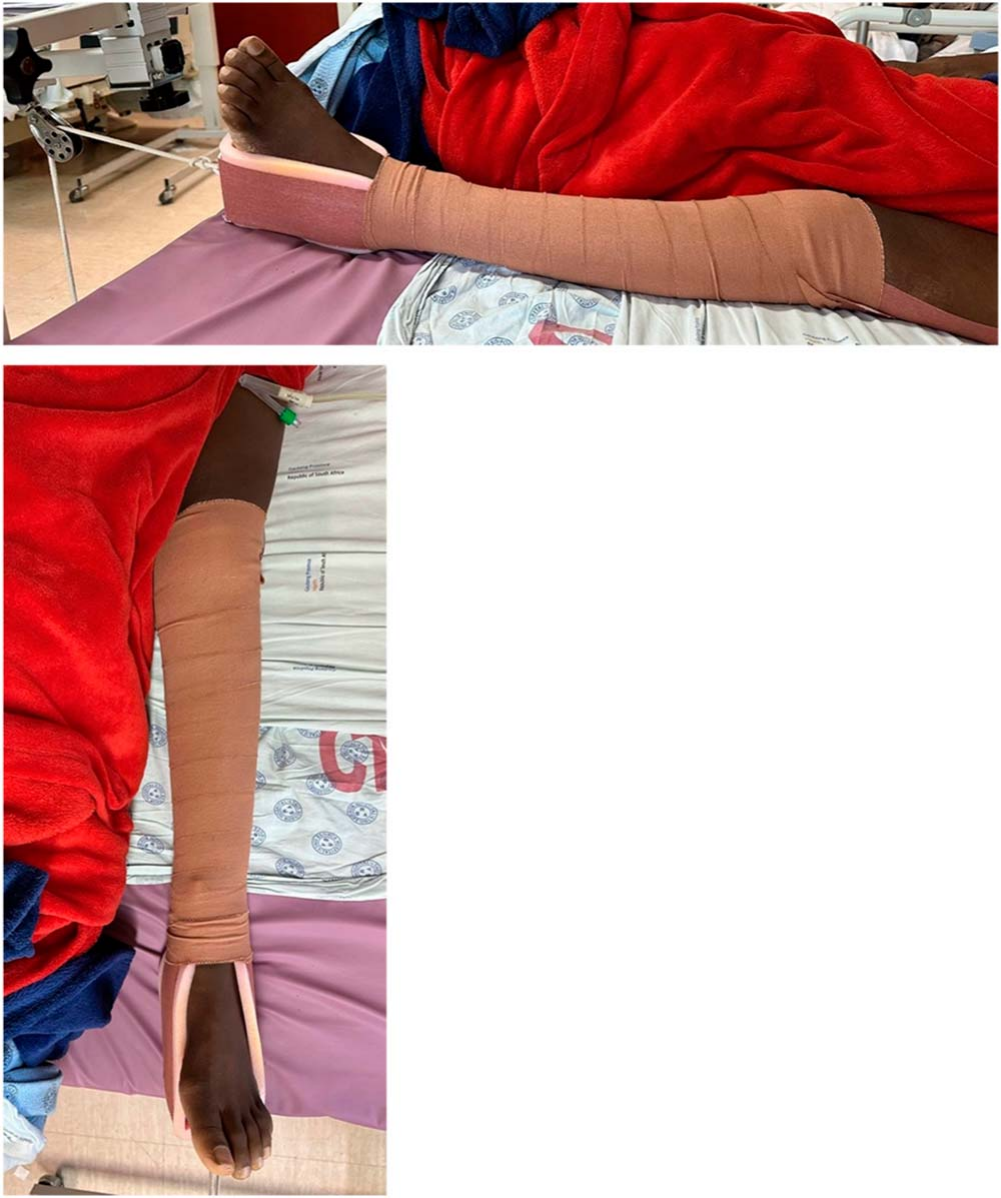
Correct application of skin traction to the leg of a patient with FNF.

There are two main types of traction potentially employed in the acute presentation of FNFs: skin and skeletal traction. Skeletal traction is invasive in application and is associated with difficulty with nursing (12), bedpan, or pressure care and has been associated with skin damage (21) due to mechanical shearing or ischaemia to the limb (10). Subsequently, skin traction stands out as the most prevalent and widely employed method of traction (22). Its application is common for the transient management of femoral neck and shaft fractures, as well as post-reduction of native hip dislocation (15). The procedure involves the non-adhesive tape on both sides of the affected limb, with careful padding over pressure areas. It is essential to avoid bandaging the malleoli and Achilles tendon. Maintaining approximately four fingers breadth slack from the sole of the foot allows for unrestricted dorsi- and plantar flexion before firm bandaging with a crepe bandage. Notably, the knee remains unbandaged to facilitate visual assessment of leg alignment. The traction apparatus is securely fastened to the bed frame, utilizing weight approximately 10% of body weight (15). Figure 2 shows the correct application of skin traction.

### Advantages and disadvantages of skin traction

In orthopaedic terms, traction is defined as the application of a pulling force to achieve the desired purpose (23) and may be applied to the arms, legs, spine, skull, or pelvis to treat fractures, dislocations, and muscle spasms and to prevent or correct deformities (15). The application of skin traction has been used for years with the principle of aligning fractured ends by maintaining continuous isotonic traction at a point distal to the fracture (23). The principle and main goal of skin traction is to control pain by relieving muscle spasm, by providing a traction and counter-traction force in the opposite direction (15). Most surgeons believe that the main theoretical advantage of the application of skin traction is to reduce and relieve pain at the fracture site until definitive surgery (9).

A secondary advantage is that by reducing the fracture with skin traction before the definitive procedure, may facilitate an easier reduction during surgery. However, this benefit is theoretical and less certain (10).

The advantages of skin traction for FNFs fractures include its relative cost-effectiveness, accessibility, minimal interference, and location to the fracture site (24). The uses of skin traction include pain relief following muscle spasm, by resting and maintaining the limb in a position of comfort, restoration of alignment following fracture, and restoration of blood flow and nerve function (23). Disadvantages include potential difficulty with nursing care (25), in terms of sanitary and pressure care, and reported damage to the skin (26) from mechanical shearing, ischaemia from tight bandages, and allergy to the adhesive strapping (9, 22, 25). Other potentially devastating complications, such as venous thromboembolism (12) from prolonged immobilization (27), decubitus ulcers, atelectasis, and pneumonia (23), have also been mentioned in the literature. Furthermore, excessive traction may result in significant arterial spasm and neuropraxia from over-stretching of neurovascular structures (23).

### Skin traction an effective means of alleviating pain?

Under the premise that in-line traction assists in fracture reduction, immobilization, and alleviating muscle spasm, the use of skin traction for FNFs is routinely used in many centres (24). In a randomized prospective control trial to determine the effects of skin traction in (28), 252 patients awaiting surgery for FNFs were allocated randomly to be nursed free in bed or to receive skin traction until surgical management. They stated that no benefit was obtained in terms of pain suffered, analgesic requirement, and/or frequency of pressure sores between the traction group and non-traction group and further stated that the routine use of skin traction for upper FNFs should be discontinued (28).

Similarly, in a separate randomized study (29), 120 patients awaiting surgery were randomized into two groups: one allocated to receive skin traction and one group without skin traction. No significant differences were found in terms of supplementary analgesic requirement between the two groups. Furthermore, radiographic follow-up at 1 week and 4 months post-surgery revealed no positive effect of avoiding avascular necrosis of the femoral head with supposed fracture reduction with the application of skin traction at time of injury (29). They further stated that preoperative skin traction use for patients with hip fractures is not recommended (29).

In both studies, pain was evaluated subjectively using the visual analogue scale (VAS), where 0/10 is regarded as pain-free and 10/10 as worse pain. Interestingly, both studies revealed that there was no difference in pain outcomes between the application of skin traction as opposed to without skin traction (28, 29), and both studies suggested that the routine use of skin traction for FNFs is not recommended.

Similar results were replicated in a randomized control study by Hussain *et al.* (30), in which 100 patients presenting with hip fractures were randomly allocated into groups with skin traction and without skin traction prior to undergoing surgery. Of their sample, 44% sustained neck of femur fractures, 33% pertrochanteric fractures, and 23% subtrochanteric fractures. Preoperative VAS scores were used statistically to determine pain relief between the two groups, concluding that between the two groups, the VAS scores in terms of pain relief were statistically not significant (30).

However, in a study by Shaik *et al.*, who compared mean pain scores recorded 24 h after the application of skin traction and a second score few minutes prior to receiving surgery, it was noted that the mean pain score was significantly reduced during the first 24 h of application of skin traction, as compared to those without skin traction, but there was no significant effect on pain beyond 24 h of application (11).

In a single-institution prospective randomized controlled study by Endo *et al.*, which included 81 patients randomized to be treated with skin traction or bed rest, findings concluded that the application of skin traction had no effect on pain relief or fracture reduction prior to receiving surgery and went on further to conclude that the application of skin traction had no effect on reduction of fracture irrespective of perioperative waiting time (9).

Furthermore, the ideal position of hip comfort for a FNF in which the intra-capsular pressure is minimized is a hip position that is slightly flexed, abducted, and externally rotated. This is contrary to the hip position for a limb immobilized in skin traction where the hip position is neutral, thereby potentially increasing intra-capsular pressure. In a study by Munireddy *et al.*, 100 patients older than 60 years of age were randomized into two groups: one group without skin traction with a pillow placed under the injured leg and one group immobilized in skin traction. Pain scores were evaluated twice a day, following analgesic administration three times a day, and findings concluded that pillow elevation of the lower limb offered better pain relief than skin traction, with a mean pain score of 2.85 for those in the skin traction group compared to a mean pain score of 2.7 for those with pillow elevation (3).

Findings in the literature are incredibly interesting and stimulate a debate on the use of skin traction following FNFs, which we have discussed and incorporated into the following table. Supplementary Table 1 (see section on Supplementary materials given at the end of the article) identifies the available literature and the recommendations for the use of skin traction in the acute management of FNFs.

### Skin traction and its risk of deep skin wounds and skin sloughing

Increasing age has profound effects on the skin, and ageing affects all components of skin (14). Most notably, thinning of the epithelium occurs with thickening of the epidermis. It is documented that both intrinsic and extrinsic factors contribute to changes in the skin of ageing individuals. Intrinsic factors, which are inherent to the biological ageing process, occur universally across all individuals. In contrast, extrinsic factors, particularly those related to environmental exposures such as prolonged ultraviolet light exposure, significantly exacerbate and accelerate the degenerative changes that occur naturally within the skin (14). These external influences compound the intrinsic ageing processes, leading to more pronounced and premature signs of skin ageing. Ultraviolet light primarily affects the dermis, decreasing the number of fibroblasts, collagen, and elastin in the skin. With the loss of collagen, and fragmentation of elastin, skin loses its tensile strength and is more prone to tearing (14).

In addition to natural ageing and the detrimental environmental effects on the skin of the elderly, venous disease is also more common in older individuals. Venous leg ulcers are associated with venous insufficiency and venous hypertension (26). Venous hypertension also leads to the manifestation of chronic venous eczema, which presents as excessive dryness and scaling of the skin, making the skin excessively fragile and prone to further breakdown and injury.

**Figure 3:**
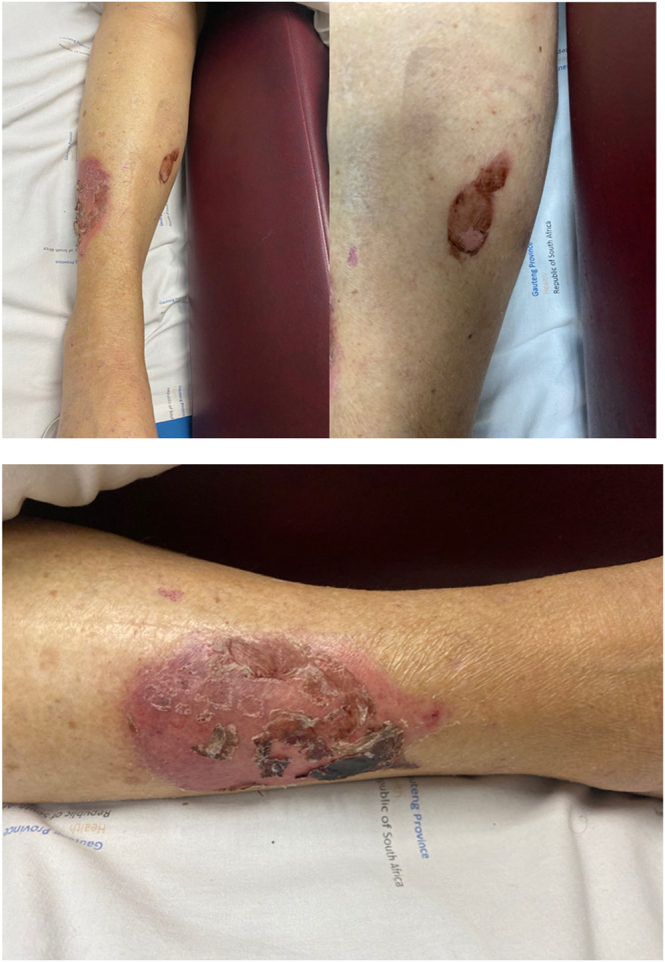
Deep skin wounds with sloughing in an elderly patient with a FNF that had adhesive skin traction application.

Shabat *et al.* identified severe skin sloughing, which they defined as an acute onset of full-thickness skin loss involving destruction of the subcutaneous tissue not extending beyond the fascia, in 10 patients with hip fractures following the application of skin traction by an orthopaedic surgeon or skilled orthopaedic nurse (25). A further 17 patients had superficial skin sloughing and were not included in their study, as they decided that only severe skin sloughing had the potential to cause major complications. Following their conclusion, they recommended that if a patient is suspected of having a complication of serious skin sloughing, notably frail skin or existing peripheral vascular disease, the skin traction device should not be used (25). Figure 3 shows deep skin wound with sloughing in an elderly patient with a FNF that had adhesive skin traction applied at our centre.

Therefore, the use of skin traction carries an increased risk of skin wounds from the adhesive, which raises concerns about the potential and theoretical risk of open leg wounds to become active cutaneous or deep tissue infection and the potential of spreading to the surgical site. Active cutaneous and deep tissue infections are documented risk factors for the devastating complication of periprosthetic joint infection in 1% of patients following hip arthroplasty (31).

Supplementary Table 2 identifies the skin-related complications following the application of skin traction in geriatric patients with FNFs and their proposed recommendation with regard to its usage.

## Conclusion

**Figure 4:**
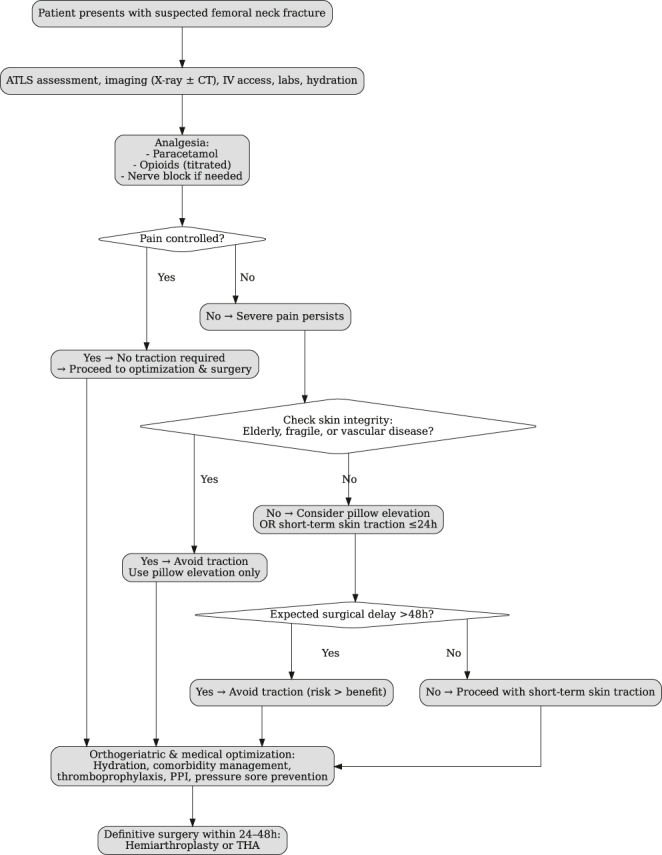
Proposed treatment algorithm: FNFs awaiting arthroplasty.

Drawing from the existing literature, and the lack thereof of centre guidelines and protocols for the usage of skin traction and documented evidence that reveals a lack of discernible benefits in terms of effective pain relief for patients with FNFs alongside the elevated risk of skin wounds and sloughing, especially in the elderly population, we have proposed the following treatment algorithm to assist with initial management of patients awaiting arthroplasty following FNFs (Fig. 4). In addition, considering the associated risk of contiguous periprosthetic joint infection following arthroplasty, a decision has been made to refrain from the routine application of skin traction for patients with FNFs awaiting arthroplasty within our centre.

## Supplementary materials





## ICMJE Statement of Interest

All authors declare that there is no conflict of interest that could be perceived as prejudicing the impartiality of the research reported.

## Funding Statement

This research did not receive any specific grant from any funding agency in the public, commercial, or not-for-profit sector.
