# Supplementary material for: The routine use of skin traction in patients with femoral neck fractures awaiting arthroplasty: a narrative review
Source: EFORT Open Rev. 2026 Mar 2;11(3):183–90. doi: 10.1530/EOR-2024-0149 (PMC12974732; doi:10.1530/EOR-2024-0149)
Supplement: Supplementary file 1 [file supplementary_table_1.pdf]

List of Tables:

Table 1: Literature review of the recommendations for application of skin traction in the acute management of neck of femur fractures.

<u>Name of Study</u>	<u>Year</u>	<u>Population Description</u>	<u>Outcome Measure</u>	<u>Results</u>
Keeping the Traction on in Orthopaedics.(1)<br><br>Choudhry B, Leung B, Filips E, Dhaliwal K.<br><br>**Cureus**	2020	Review article<br><br>Great Britain<br><br>No participants included<br><br>Describes the use of skin traction, Hamilton-Russel traction.<br><br>(Not specific for FNFs)	No intervention<br><br>Review of the literature and description of various techniques used (skin vs skeletal vs manual traction.	Concluding remarks in article: *traction in general* 'remains an important technique for pain relief, keeping long bone fractures reduced and preventing joint contractures.'<br><br>Complications mentioned: common peroneal nerve neuropraxia, pressure sores.
Current Place of Traction in Orthopaedic and Trauma Practice : A Review.(2)<br><br>Obalum D, Ibeanusi S.<br><br>**Orthop Rheumatol**.	2019	Review article<br><br>Nigeria<br><br>Literature review<br><br>No participants included<br><br>(Not specific for FNFs)	No intervention<br><br>Also reviews literature and gives description of various techniques used (skin vs skeletal vs manual traction.<br><br>Listed benefits: cost-effective, pain reduction, prevent muscle spasm<br><br>Mentioned a Cochrane review on pre-op traction for FNF's which showed <u>no benefit</u> for patients.<br><br>*Handoll HH, Queally JM, Parker MJ. Pre-operative traction for hip fractures in adults. Cochrane Database Syst Rev. 2011 Dec 7;(12)*	Concluding remarks in article: 'Traction still has some role in current practice particularly in resource-challenged regions as well as in the developed world especially as temporary treatment, pre, intra, and postoperative periods in orthopaedics and trauma. However, traction should not be adopted as the standard of care where better options of care are available.'
The effectiveness of skin traction	2021	Systematic review: Search of	Does the use of pre-operative skin traction reduce pain in	"The results confirm those of earlier reviews:

in reducing pain in adults with a hip fracture: A systematic review. (3)<br><br>Sammut R, Attard M, Mangion D, Trapani J.<br><br>**Int J Orthop Trauma Nurs.**		PubMed/Medline etc<br><br>Malta<br><br>Pre-operative skin traction for FNF's<br><br>Five RCT's included (between 2011 and 2020)	patients with FNF's?<br><br>Pain scores (VAS and analgesic requirements)<br><br>Four of the five studies included in this review reported that pre-operative skin traction had no effect on pain relief prior to surgery, irrespective of the preoperative waiting time	skin traction does not significantly decrease pain and analgesia use. The quality of the evidence available is weak. There is no strong evidence for the routine pre-operative use of skin traction in adults with a hip fracture."<br><br>Remarks re complications: "use is controversial due to a lack of evidence to its benefits and possible complications such as pressure ulcers, nerve damage, VTE. "<br><br>This means that traction should be discouraged as standard practice as there are no favorable effects.
Why are we still using pre-operative skin traction for hip fractures? (4)<br><br>Yip D, Chan C, Chiu PK, Wong JW, Kong JK.<br><br>**Int Orthop.**	2002	Hong Kong<br><br>Prospective randomized trial<br><br>311 patients with FNF's<br><br>Two groups (2kg traction vs pillow under knee)<br><br>205 woman/106 men – average age 79<br><br>4.7 days average time to surgery.<br><br>Hemi/DHS/Cannulated screws included.	VAS score 4 times per day<br><br>Analgesic dispensing	A significant difference in pain score on the evening of admission and the first morning after admission between the groups with traction compared the group without was found. However, there was no corresponding increase in analgesic requirement during this period.<br><br>Conclusion: No other objective benefit can be shown from using skin traction, and its routine use should be abandoned.

				Complications mentioned: It makes nursing the patient more difficult; for example, lifting the patient onto a bedpan or in pressure area care prior to surgery. Damage to the skin by mechanical shearing, ischemia to the limb from tight bandages, or allergy to adhesive strapping.
Efficacy of Skin Traction in Elderly Neck of Femur Fractures for Pre Operative Pain Relief. (5)<br><br>Munireddy DS, Chandra DS, Senthil DL.<br><br>**IOSR J Dent Med Sci.**	2016	India<br><br>100 elderly patients (37 Men, 63 Women)<br><br>Age >60 years, average 67<br><br>FNF's<br><br>Pillow under leg vs skin traction (6% body weight) groups<br><br>Young patients/PVD/Skin conditions excluded.	VAS score BD<br><br>Drug dispensation TDS	The peak pain score was found to be on the end of day one for both groups after which we saw a decline in the pain scores of both groups.<br><br>The p value for difference in pain score and analgesic requirements was not less than 0.05 and hence statistically insignificant.<br><br>Conclusion: 'in our study, skin traction did not result in significant pain relief in elderly neck of femur fracture patients.
The usefulness of preoperative traction in hip fracture (6).<br><br>Foronda IE, Caeiro-Rey JR. Rev	2018	Spain<br><br>Literature review (PubMed/SCOPUS/EMBASE)<br><br>Key words; 'Hip fracture, pre-op, skin traction'<br><br>Randomized prospective studies selected.<br><br>14 studies<br><br>Skin traction vs pillow	No intervention<br><br>Review of the literature<br><br>Also mention Cochrane review 2011<br><br>Intensity of pain	No benefit of skeletal over skin<br><br>No difference in pain scores with traction vs without across ALL 14 studies.<br><br>Conclusion: skin traction in patients with

**Osteoporos y Metab Miner.**		Also looked at skin vs skeletal traction	Need for analgesics	hip fractures waiting to be operated on should be eliminated from routine practice. There is no evidence to support that their supposed benefits, the improvement of pain and a better reduction of the fracture that facilitates the surgery, are real.
Pre-operative traction for hip fractures in adults. (7)<br><br>Handoll HH, Queally JM, Parker MJ.<br><br>**Cochrane Database Syst Rev.**	2011	UK based<br><br>1997/2006/2011 updates<br><br>Traction, pre-operative, FNF's<br><br>Skin vs no skin (10 trials) AND skin vs skeletal (1 trial)<br><br>Medline/EMBASE/Cochrane library and special register.<br><br>Focused on RCTs<br><br>11 trials, 1654 patients.	VAS scores for pain.<br><br>Analgesia use<br><br>Presence of pressure ulcers<br><br>Ease to reduce fracture (subjective surgeon opinion)<br><br>Incidence of fracture healing complications<br><br>Amongst others.	No evidence of benefit from traction either in the relief of pain, ease of fracture reduction or quality of fracture reduction at time of surgery.<br><br>Inconclusive evidence for pressure sores. Sensory changes and blisters were reported.<br><br>Skeletal traction was more painful and costly.<br><br>Conclusion: 'Routine use of traction does not appear to have any benefit'.
Preoperative traction for hip fracture. A randomized comparison between skin and skeletal traction in 78 patients.(8)<br><br>Resch S, Thorngren KG.<br><br>**Acta Orthop Scand.**	1998	Sweden<br><br>Skin vs skeletal traction<br><br>78 patients (75 excluded for confusion)<br><br>Cervical and Trochanteric femur fractures<br><br>Average age 81	VAS/Number of doses of analgesia	No difference in VAS score<br><br>Application of skeletal traction found to be painful in 50% of patients<br><br>Skeletal traction should not be used in these patients pre-operatively.

Efficacy of preoperative skin traction for hip fractures: A single-institution prospective randomized controlled trial of skin traction versus no traction. (9)<br><br>Endo J, Yamaguchi S, Saito M, Itabashi T, Kita K, Koizumi W, et al.<br><br>**J Orthop Sci.**	2013	Japan<br><br>Prospective randomized control trial<br><br>Skin traction vs no traction<br><br>Efficacy of traction when surgery is delayed<br><br>81 patients (41 traction/40 no traction)<br><br>Mean time to surgery 7.5 days<br><br>28 excluded because of senility.<br><br>Femoral neck and trochanteric fractures included.	VAS/Number of doses of analgesia<br><br>Fracture reduction based on leg length, neck shaft angle on admission/pre/post op.<br><br>Complications: looked for erythema, blisters, DVT, Pneumonia, 1 year mortality.	No difference in pre-op pain (vas or number of analgesics) between groups.<br><br>No significant difference in radiographic data before or after surgery.<br><br>Conclusion: no change in pain. No change in fracture reduction during surgery.<br><br>Incidence of complications was higher in the traction group.
Preoperative position splint versus skin traction in patients with hip fracture: An experimental study. (10)<br><br>Tosun B, Aslan O, Tunay S.<br><br>**Int J Orthop Trauma Nurs**	2018	Turkey<br><br>Randomized trial<br><br>Position splint vs skin traction (control group)<br><br>Hip fractures (including sub and intertroch)<br><br>68 participants (50% split)	Pain (VAS)<br><br>Comfort (Immobilization comfort questionnaire)<br><br>Complications (pressure sores using Braden scale)<br><br>Patient satisfaction<br><br>Introduction mentions potential complications of traction e.g. VTE, pressure ulcers, delirium, nerve damage.	Preoperative position splint application in patients with hip fracture relieved pain and complications and increased comfort and satisfaction with treatment and care.<br><br>Skin traction group saw more complications: Constipation, pressure ulcers and adhesive plaster allergy.
Pain relief differentiated according to the length of time that preoperstive skin traction was carried out	2021	Japan<br><br>Systematic review and Meta-analysis<br><br>PubMed, Google Scholar, Cochrane Library, Web of Science and MEDLINE search<br><br>Comparison of pain relief between preoperative skin traction and no traction for	VAS scores at 1 hour, 4-6 hours, 12, and 24 hours postadmission	Preoperative skin traction can provide pain relief within 1 hour postadmission, but not at 4-6, 12, and 24 hours

for hip fractures: A systematic review and meta-analysis (11)<br><br>Kobayashi T, Ureshino H, Morimoto T, Sonohata M, Mawatari M.<br><br>**Int J Orthop Trauma Nurs**		hip fracture<br><br>Five studies involving 413 patients		
Comparison of mean pain score by applying with and without skin traction in patients with hip fracture (12).<br><br>Hussain Z, Raza S, Momin A, Ali N.<br><br>**Pakistan J Med Heal Sci.**	2015	Pakistan<br><br>Randomized trial<br><br>Skin traction vs no traction<br><br>100 patients (50/50)<br><br>Mean age 39<br><br>Included FNF, intertroch, subtroch<br><br>68% male	VAS scores	The conclusion of the study is that routine use of skin traction in patients with hip fractures has no effect in reduction of pain preoperatively.
Clinical practice and nursing management of pre-operative skin or skeletal traction for hip fractures in elderly patients: a cross-sectional three-institution study. (13)<br><br>Biz C, Fantoni I, Crepaldi N,	2019	Italy<br><br>Cross-sectional study<br><br>12 item survey given to nurses/surgeons<br><br>136 surveys<br><br>FNF including inter and subtroch.<br><br>Literature review also done.	Staffs perception of indications and complications for skin and skeletal traction.	Mainly applied for subtroch fractures.<br><br>Findings: Pain management, VTE and pressure ulcer prevention were perceived as worse only with skeletal traction, while hygiene was described as more difficult with both skeletal and skin traction.

Zonta F, Buffon L, Corradin M, et al.<br><br>**Int J Orthop Trauma Nurs**				Comments:<br><br>Skin traction is still in use despite little indication – perhaps this is a cultural element from older surgeons.<br><br>Skin traction may worsen pain, increase nursing difficulty, worsen hygiene, increase risk for VTE and hamper pressure ulcer prevention.<br><br>Skin traction for proximal femur fractures should be discouraged.
Hip Fracture: Comparison of mean pain score between skin traction versus without skin traction in cases presenting. (14)<br><br>Shaikh A, Shahwani M, Ishaq M.<br><br>**Prof Med J.**	2018	Pakistan<br><br>Randomized trial<br><br>Unilateral femur fracture within 72 hours admission<br><br>Skin traction vs no traction<br><br>Mean age 48<br><br>They did not declare whether these were only FNF, if they included intertroch and subtroch.	VAS score after 24 hours and a few hours prior to surgery.	Mean pain score is significantly reduced during first 24 hours of application of skin traction as compared to those without it in cases with hip fractures, however, it has no significant effect on pain after 24 hours of application.
An Explorative Study to Find Out the Prevalence of Complication among Orthopaedic Patients with	2019	India<br><br>No description of what fractures were included<br><br>All forms of skin and skeletal traction?<br><br>Quantitative research	Prevalence of complications in each group.	Slightly higher overall complications in skeletal vs skin traction group.<br><br>They did not define what these complications were.

Skin Traction and Skeletal Traction. (15)<br><br>Dkhar MP, Sengupta PM, Iawim MR.<br><br>**International journal of health sciences and research.**		No intervention<br><br>60 patients		
Preoperative skin traction for fractures of the proximal femur. A randomised prospective trial (16)<br><br>Anderson GH, Harper WM, Connolly CD, Badham J, Goodrich N, Gregg PJ.<br><br>**J Bone Jt Surg**	1993	GBR<br><br>Randomized control trial<br><br>252 patients awaiting surgery<br><br>Skin traction vs no traction<br><br>Mean age 80.6<br><br>Intra and extracapsular fractures<br><br>Remark that little evidence for pain relief and fracture position improvement exists.<br><br>Risks mentioned: mechanical shearing of skin, adhesive allergy, difficult nursing care, VTE risks.	VAS TDS<br><br>Analgesic doses given<br><br>Pressure score grading against photograph references<br><br>Mental test score<br><br>Waterlow score for risk of pressure sores	No differences were found between the groups in terms of pain suffered, analgesia required, frequency of pressure sores or ease of operation.
Preoperative skin traction in patients with hip fractures is not useful. (17)<br><br>Jerre R, Doshé A, Karlsson J.<br><br>**Clin Orthop**	2000	Sweden<br><br>120 participants<br><br>Prospective randomized control trial<br><br>Skin traction vs no traction pre op<br><br>Cervical and Trochanteric groups	Pain<br><br>Need for analgesics<br><br>Complications<br><br>Quality of reduction	Skin traction has no benefits<br><br>Its use should be abandoned.<br><br>No difference in pain scores or reductions.<br><br>Complications seen only in the traction groups.

**Relat Res.**				
The use of skin traction in the adult patients with proximal femur fracture. What are the effects, advantages and disadvatages? A scoping review (18)<br><br>Miedico M, Quattrini F, Attardo SE, Marchioni M, Bassi MC, Lucenti E, et al.<br><br>**Int J Orthop Trauma Nurs**	2023	Italy<br><br>Literature review (PubMed/CINAHL/COCHRANE/Embase/DOAJ/<br>ClinicalTrial.gov/OpenDissertation)<br><br>Clinical question: 'which are the effects of skin traction, its advantages and disadvantages in adult patients with proximal femur fractures hospitalized in orthopaedic wards?<br><br>9 records included.<br><br>Skin traction effects summarized in 7 categories: pain, pressure sores, comfort and relaxation, thromboembolism, damage from adhesive, complications, and quality of care.	No intervention<br><br>Review of the literature and description of various effects of skin traction application in patients with proximal femur fractures	Routine use of skin traction not recommended.<br><br>Suggest multicenter studies focused on producing more consistent evidence for use such as decrease in pain within 24h – 60h post admission.

Bibliography:

1. Choudhry B, Leung B, Filips E, Dhaliwal K. Keeping the Traction on in Orthopaedics. Cureus. 2020;12(8):1–24.
2. Obalum, Ibeanusi. Current Place of Traction in Orthopaedic and Trauma Practice : A Review. Orthop Rheumatol [Internet]. 2019;13(5):1–4.
3. Sammut R, Attard M, Mangion D, Trapani J. The effectiveness of skin traction in reducing pain in adults with a hip fracture: A systematic review. Int J Orthop Trauma Nurs.
4. Yip D, Chan C, Chiu PK, Wong JW, Kong JK. Why are we still using pre-operative skin traction for hip fractures? Int Orthop. 2002;26(6):361–4.
5. Munireddy DS, Chandra DS, Senthil DL. Efficacy of Skin Traction in Elderly Neck of Femur Fractures for Pre Operative Pain Relief. IOSR J Dent Med Sci. 2016;15(08):15–7.

6. Foronda IE, Caeiro-Rey JR. The usefulness of preoperative traction in hip fracture. Rev Osteoporos y Metab Miner. 2018;10(2):98–102.
7. Handoll HH, Queally JM, Parker MJ. Pre-operative traction for hip fractures in adults. Cochrane Database Syst Rev. 2011.
8. Resch S, Thorngren KG. Preoperative traction for hip fracture. A randomized comparison between skin and skeletal traction in 78 patients. Acta Orthop Scand. 1998;69(3):277–9.
9. Endo J, Yamaguchi S, Saito M, Itabashi T, Kita K, Koizumi W, Kawaguchi Y, Asaka T, Saegusa O. Efficacy of preoperative skin traction for hip fractures: A single-institution prospective randomized controlled trial of skin traction versus no traction. J Orthop Sci.
10. Tosun B, Aslan O, Tunay S. Preoperative position splint versus skin traction in patients with hip fracture: An experimental study. Int J Orthop Trauma Nurs.
11. Kobayashi T, Ureshino H, Morimoto T, Sonohata M, Mawatari M. Pain relief differentiated according to the length of time that preoperative skin traction was carried out for hip fractures: A systematic review and meta-analysis. Int J Orthop Trauma Nurs.
12. Hussain Z, Raza S, Momin A, Ali N. Comparison of mean pain score by applying with and without skin traction in patients with hip fracture. Pakistan J Med Heal Sci. 2015;9(1):185–8.
13. Biz C, Fantoni I, Crepaldi N, Zonta F, Buffon L, Corradin M, Lissandron A, Ruggieri P. Clinical practice and nursing management of pre-operative skin or skeletal traction for hip fractures in elderly patients: a cross-sectional three-institution study. Int J Orthop Trauma Nurs.
14. Shaikh AM, Shahwani MB IM. HIP fracture; comparison of mean pain score between skin traction versus without skin traction in cases presenting. Prof Med J. 2018;25(1):30–3.
15. Dkhar MP, Sengupta PM, Iawim MR. An Explorative Study to Find Out the Prevalence of Complication among Orthopaedic Patients with Skin Traction and Skeletal Traction , Admitted In Selected Hospital , Guwahati , Assam. 2019;9(October):131–5.
16. Anderson GH, Harper WM, Connolly CD, Badham J, Goodrich N, Gregg PJ. Preoperative skin traction for fractures of the proximal femur. A randomised

prospective trial. J Bone Jt Surg - Ser B. 1993;75(5):794–6.

17. Jerre R, Doshé A, Karlsson J. Preoperative skin traction in patients with hip fractures is not useful. Clin Orthop Relat Res. 2000;378(378):169–73.

18. Miedico M, Quattrini F, Attardo SE, Marchioni M, Bassi MC, Lucenti E, Sarli L, Guasconi M. The use of skin traction in the adult patients with proximal femur fracture. What are the effects, advantages and disadvantages? A scoping review. Int J Orthop Trauma Nurs.
