# Supplementary material for: The routine use of skin traction in patients with femoral neck fractures awaiting arthroplasty: a narrative review
Source: EFORT Open Rev. 2026 Mar 2;11(3):183–90. doi: 10.1530/EOR-2024-0149 (PMC12974732; doi:10.1530/EOR-2024-0149)
Supplement: Supplementary file 2 [file supplementary_table_2.pdf]

List of Tables:

Table 2: Literature review identifying skin related complications following application of skin traction.

A deep wound in left leg as a result of skin traction in 81-old-year woman with hip fracture in orthopaedic ward. (1)<br><br>Tafti AA, Sajadi SS, Rafiei H<br><br>**Int Wound J.**	2013	Iran<br><br>Case report<br><br>81 year old woman<br><br>Describes how skin traction is not without risks.<br><br>Patient sustained an intertrochanteric fracture.	N/A<br><br>Skin breakdown<br><br>Old patient<br><br>Friable skin/Poor skin perfusion due to CVS risk factors.	Advise not using skin traction in elderly patients with vulnerable skin.
Deep skin slough following skin traction for hip fractures. (2)<br><br>Shabat S, Gepstein R, Mann G, Kish B, Fredman B, Nyska M.<br><br>**J Tissue Viability.**	2002	Israel<br><br>10 patients fulfilled criteria<br><br>Age between 76-90<br><br>FNF/intertroch/subtroch included	'serious skin slough' defined as depth to subcutaneous tissue but not past fascia (not involving bone or tendon etc.)<br><br>Prevalence of chronic illness<br><br>Age	Leading chronic illness was cardiovascular disease.<br><br>Skin slough occurred during or immediately after surgery,<br><br>Treated with daily 'jelonet' type dressings.<br><br>All required outpatient follow up and dressings<br><br>Conclusion: Skin traction can cause serious skin slough which interferes with rehabilitation and may prolong hospital stay. Skin traction should be avoided if

					possible.
Management of the skin and soft tissue in the geriatric surgical patient. (3)<br><br>Greenhalgh DG.<br><br>**Surg Clin North Am.**	2015	US	Review article<br><br>Skin healing in the elderly.<br><br>Pathophysiology of skin healing in elderly<br><br>Risk factors for elderly	No outcome measures<br><br>No intervention	Aging leads to intrinsic skin changes resulting in thinning, loss of dermal appendages, and increased fragility that increase the risk of injury and impair wound healing; the aging process can be accelerated by extrinsic factors such as exposure to sunlight.<br><br>Disease processes that are more prevalent in the elderly, such as malnutrition, diabetes mellitus, treatments for malignancy, and vascular disease, all impair tissue repair<br><br>There are functional changes in aging that predispose the elderly to increased risk and an impaired ability to handle major wounds.<br><br>Chronic wounds such as pressure ulcers and venous stasis ulcers are extremely difficult to treat.
Skin considerations for older adults with wounds.(4)<br><br>Holloway S.	2019	GBR	Review article<br><br>Skin healing in the elderly.<br><br>Overview of wound healing<br><br>Main focus on chronic wound healing.<br><br>Overview of medical adhesive-related skin	No outcome measures<br><br>No intervention	“Common to all individuals with wounds requiring some sort of dressing is the risk of medical adhesive-related skin injury, where dressing application and removal need to be of the

**Br J Community Nurs.**	injury (MARSI)	utmost priority to reduce the risk of injury.”

Bibliography:

1. Tafti AA, Sajadi SS, Rafiei H. A deep wound in left leg as a result of skin traction in 81-old-year woman with hip fracture in orthopaedic ward. Int Wound J. 2013;10(4):484.
2. Shabat S, Gepstein R, Mann G, Kish B, Fredman B, Nyska M. Deep skin slough following skin traction for hip fractures. J Tissue Viability. 2002;12(3):108–12.
3. Greenhalgh DG. Management of the skin and soft tissue in the geriatric surgical patient. Surg Clin North Am. 2015;95(1):103–14.
4. Holloway S. Skin considerations for older adults with wounds. Br J Community Nurs. 2019;24(June):S15–9.
